# Genetic relatedness in carbapenem-resistant isolates from clinical specimens in Ghana using ERIC-PCR technique

**DOI:** 10.1371/journal.pone.0222168

**Published:** 2019-09-12

**Authors:** Francis S. Codjoe, Charles A. Brown, Thomas J. Smith, Keith Miller, Eric S. Donkor

**Affiliations:** 1 Department of Medical Laboratory Sciences, School of Biomedical & Allied Health Sciences, College of Health Sciences, University of Ghana, Ghana; 2 Biomolecular Science Research Centre, Sheffield Hallam University, Sheffield, England, United Kingdom; 3 Department of Medical Microbiology, School of Biomedical & Allied Health Sciences, College of Health Sciences, University of Ghana, Ghana; School of Pathology, National Health Laboratory Service (NHLS) and University of the Witwatersrand, South Africa, SOUTH AFRICA

## Abstract

**Aim:**

Enterobacterial repetitive intergenic consensus (ERIC) sequence analysis is a powerful tool for epidemiological analysis of bacterial species. This study aimed to determine the genetic relatedness or variability in carbapenem-resistant isolates by species using this technique.

**Methods:**

A total of 111 non-duplicated carbapenem-resistant (CR) Gram-negative bacilli isolates from a three-year collection period (2012–2014) were investigated by enterobacterial repetitive intergenic consensus-polymerase chain reaction (ERIC–PCR) in four selected hospital laboratories in Ghana. The isolates were also screened for carbapenemase and extended spectrum β-lactamase genes by PCR.

**Results:**

A proportion of 23.4% (26/111) of the genomic DNA extracts were carriers of PCR-positive carbapenemase genes, including 14.4% *bla*NDM-1, 7.2% *bla*VIM-1 and 1.8% *bla*OXA-48. The highest prevalence of carbapenemase genes was from non-fermenters, *Acinetobacter baumannii* and *Pseudomonas aeruginosa*. For the ESBL genes tested, 96.4% (107/111) of the CR isolates co-harboured both TEM-1 and SHV-1 genes. The ERIC-PCR gel analysis exhibited 1 to 8 bands ranging from 50 to 800 bp. Band patterns of 93 complex dissimilarities were visually distinguished from the 111 CR isolates studied, while the remaining 18 showed band similarities in pairs.

**Conclusion:**

Overall, ERIC-PCR fingerprints have shown a high level of diversity among the species of Gram-negative bacterial pathogens and specimen collection sites in this study. ERIC-PCR optimisation assays may serve as a suitable genotyping tool for the assessment of genetic diversity or close relatedness of isolates that are found in clinical settings.

## Background

The enterobacterial repetitive intergenic consensus (ERIC) technique is a molecular method used for the epidemiological analysis and genotyping of bacterial species. The application of ERIC offers a greater potential for the study of the development of bacterial interspersed repetitive arrangements because sequences are lengthier and do not rely on targeting a specific region of the genome. In addition, it offers detailed information to enable comparative analysis in a wide range of bacterial species [1, 2].

It is noteworthy that the ERIC technique has been used in many different areas of research including veterinary microbiology, food microbiology and, in particular, various clinical areas dealing with human infections within the hospital environment and in the community [3–7]. Indeed, enterobacterial repetitive intergenic consensus-polymerase chain reaction (ERIC-PCR) can be used to study human pathogens, in both Gram-positive and -negative bacterial isolates in order to determine their genetic diversity, and occasionally extended to bacterial pathogens in the animal kingdom. Further, genomic DNA fingerprints from ERIC-PCR have proved to be useful for the investigation of other organisms apart from those in the Enterobacteriaceae family such as *Aeromonas* species, [8] *Staphylococcus aureus*, [9] and *Haemophilus parasuis* [10]. More recently, the application of ERIC-PCR has been used to investigate opportunistic pathogens, including *Pseudomonas aeruginosa* and *Acinetobacter baumannii*, that are capable of causing outbreaks in hospitals worldwide [11–16]. Isolates of *Pseudomonas* spp. and *Acinetobacter* spp. can easily acquire multiple resistance to a panel of antimicrobials including carbapenems, share common genes, transfer resistant traits both intra and interspecies, and the encoded genes they carry are extremely mobile, particularly in *Pseudomonas aeruginosa* [17, 18]. Good reproducibility and applicability in the determination of close relatedness have been reported in several studies on a variety of pathogens, which share the same homogeneities. For most Gram-negative bacterial strains, diversity/similarity among pathogens have traditionally been determined using antimicrobial resistance patterns, basic microbiological methods or genotyping methods such as by gene expression using microarray technology, multilocus sequence typing (MLST), pulsed field gel electrophoresis (PFGE) and ERIC. To date, there are no reports on the investigation of the presence of β-lactamase determinants in carbapenem-resistant (CR) isolates and their clonal relatedness or variabilities in Ghana. This study aimed to broadly determine the genetic relatedness or variability in both carbapenemase PCR-positive isolates and CR isolates without a genetically identified locus (carbapenemase PCR negative), and separately assess clonal similarities for all the PCR-positive species using fingerprint patterns generated by ERIC-PCR amplifications.

## Methods

### The study isolates

The study isolates comprised 111 non-duplicated CR Gram-negative bacilli isolates collected from four selected hospital laboratories in Ghana over a three-year period (2012–2014). The hospitals included Effia-Nkwanta Hospital (ENH) in the Western Region, AngloGold Mines Hospital (AMH) in the Ashanti Region, Ho Regional Hospital (HRH) in the Volta Region and Korle-Bu Teaching Hospital in the Greater Accra Region (KBTH). These hospitals were selected to represent the different types of hospitals in Ghana including regional hospitals (HRH and ENH), a district hospital (AMH) and in a tertiary hospital (KBTH). The CR isolates consisted of 51 *Pseudomonas aeruginosa*, 31 *Acinetobacter baumannii*, 12 *Escherichia coli*, 7 *Pseudomonas putida*, 3 each of *Klebsiella pneumoniae* and *Enterobacter cloacae*, and one each of *Cronobacter sakazakii*, *Providencia stuartii*, *Shigella sonnei* and *Sphingomonas paucimobilis*. The organisms were isolated from ten specimen types but most of the isolates were from wound infections (47) and urinary specimens (31). The Vitek 2 automated compact system (BioMérieux, France) was used to identify the CR isolates to species level. *Escherichia coli* ATCC 25922, which is susceptible to carbapenems, and *Klebsiella pneumoniae* carbapenemase positive NCTC 13438 were included in the identification as controls. Carbapenem resistance of the CR isolates was determined by both disc diffusion test and E-test.

### PCR analysis of carbapenemase and extended spectrum β-lactamase genes

DNA extraction of the CR isolates was performed using the QiaAmp mini Kit (Qiagen, Hilden, Germany). The DNA samples were used in PCR analysis of carbapenemase and extended spectrum β-lactamase genes. The carbapenemase genes screened were Oxacillinase-48 (OXA-48), New Delhi metallo-beta-lactamase-1 (NDM-1), Imipenem-resistant *Pseudomonas*-1 (IMP-1), Verona integron-encoded metallo-β-lactamase-1 (VIM-1), and *Klebsiella pneumoniae* carbapenemase (KPC). The ESBL genes screened were *bla*TEM and *bla*SHV. The PCR reaction mix was aseptically prepared using PyroMark Master Mix Kit (Qiagen, Hilden, Germany). PCR amplification of carbapenemase and ESBL genes were based on primers described by Poirel *et al*. [19] and Schlesinger *et al*. [20] respectively. These primers as well as the PCR cycling conditions are reported in Table 1.

**Table 1:** Primer sets for amplification of carbapenemase and extended spectrum β-lactamase genes.

**Gene**	**Primer sequence (5’→3’)**	**Amplicon size (bp)**	**PCR cycling conditions**	**Reference**
*bla*IMP	Forward—GGAATAGAGTGGCTTAAYTCTC Reverse–GGTTTAAYAAAACAACCACC	232	Initial denaturation at 95°C for 3 minutes, followed by 40 cycles of denaturation at 95°C for 1 minute, annealing at 58°C for 30 seconds, and elongation at 72°C for 1 minute 30 seconds, followed by a final elongation step at 72°C for 10 minutes	Poirel *et al*. [19]
*bla*VIM	Forward—GATGGTGTTTGGTCGCATA Reverse—CGAATGCGCAGCACCAG	390		Poirel *et al*. [19]
*bla*OXA-48	Forward—GCGTGGTTAAGGATGAACAC Reverse–CATCAAGTTCAACCCAACCG	438		Poirel *et al*. [19]
*bla*NDM	Forward—GGTTTGGCGATCTGGTTTTC Reverse–CGGAATGGCTCATCACGATC	621		Poirel *et al*. [19]
*]bla*KPC	Forward—CGTCTAGTTCTGCTGTCTTG Reverse–CTTGTCATCCTTGTTAGGCG	798		Poirel *et al*. [19]
*bla*TEM	Forward—TCAACATTTTGTCGTCG Reverse–CTGACAGTTACCAATGCTTA	860	Initial denaturation 15 minutes at 95°C and 35 cycles of 1 minute at 94°C, 1 minute at an annealing temperature of 47°C and 50°C designed for each primer set for TEM and SHV respectively, and 1 min at 55°C, followed by 10 minutes at 72°C for the final extension.	Schlesinger *et al*. [20]
*bla*SHV	Forward—TTTATCGGCCYTCACTCAAGG Reverse–GCTGCGGGCCGGATAACG	930		Schlesinger *et al*. [20]
ERIC	Forward- AAGTAAGTGACTGGGGTGAGCG Reverse-ATGTAAGCTCCTGGGGATTCAC		Reaction conditions were: 95°C for 15 minutes and 45 cycles of 94°C for 30 seconds, 45°C for 45 seconds, and 72°C for 7 minutes, followed by a final extension at 72°C for 10 minutes	Ye *et al*. [21]

Key: IMP, imipenem-resistant *Pseudomonas*; VIM, Verona integron-encoded metallo-β-lactamase; OXA-48, oxacillinase-48; NDM, New Delhi metallo-β-lactamase; KPC, *Klebsiella pneumoniae* carbapenemase; TEM-1, Temoniera-1; SHV-1, sulphydry1 variable-1; ERIC, Enterobacterial repetitive intergenic consensus.

### Enterobacterial repetitive intergenic consensus-PCR (ERIC-PCR)

Enterobacterial repetitive intergenic consensus-PCR (ERIC-PCR) was performed using the DNA extracts and reaction mixture described in 2.2. The primers and cycling conditions used for the ERIC-PCR were those described by Ye *et al*. [21], which are also illustrated in Table 1. Band patterns obtained by ERIC-PCR were visually evaluated in the absence of appropriate software. All isolates were analysed with duplicate gels in each electrophoresis run for uniformity. For quality control and consistency in DNA migration during electrophoresis, all the gels were electrophoresed for an equal period of time. Isolates with two or more different bands were interpreted as unrelated.

### Fingerprints of ERIC-PCR

Dendrograms were produced using the positions of the band lanes on each agarose gel normalise against a standard 1 kb DNA molecular marker as a reference. The location of each given band was located as one and no band as zero. The nearest band patterns of each bacterial species were used to analyse the similarity or variability matrix calculated by the number of base differences. Dendrograms of ERIC-PCR fingerprint patterns were assembled for both PCR carbapenemase negatives and carbapenemase positive gene carriers together and separately for all carbapenemase positive gene carriers based on each species. This was done with the aid of Gel ComparII image analysis software (version 6.6.11, Applied Maths, Kortrijk, Belgium), and the unweighted pair group method with arithmetic mean (UPGMA) cluster method was applied to all data.

### Ethical considerations

The study was approved by the Ethical Committee of the School of Biomedical and Allied Health Sciences, University of Ghana (Ethics Identification Number: SAHS-ET/SAHS/PSM/ML/05/AA/26A/2012-2013). As the samples used in the study were archived isolates, we could not obtain patients’ consent for use of their clinical data. However, all patients’ data and isolates were de-identified to ensure anonymity.

## Results

### 3.1 Genotypic assays of CR isolates

Genotyping by PCR assay identified 26/111 (23.4%) of the genomic DNA extracts as carriers of PCR-positive carbapenemase genes, including 14.4% blaNDM-1, 7.2% blaVIM-1 and 1.8% blaOXA-48 genes. The distribution of carbapenemase genes in the Gram-negative study isolates were as follows: *Acinetobacter baumannii* (9 NDM-1 positives); *Pseudomonas aeruginosa* (2 NDM-1 and 7 VIM-1); *Escherichia coli* (3 NDM-1); *Klebsiella pneumoniae* (2 OXA-48); *Pseudomonas putida* (1 VIM-1); *Providencia stuartii* (1 NDM-1); and *Shigella sonnei* (1 NDM-1). For the ESBL genes tested, 96.4% (107/111) of the CR isolates co-harboured both TEM-1 and SHV-1 genes. However, three of the isolates that were negative on the PCR assay harboured the TEM-1 gene alone. All of the 26 carbapenemase-positive gene carrying isolates harboured both the ESBL genes (TEM-1 and SHV-1) except the *Shigella sonnei* strain which harboured only the TEM-ESBL gene.

### 3.2 ERIC-PCR analysis

**Fig 1:**
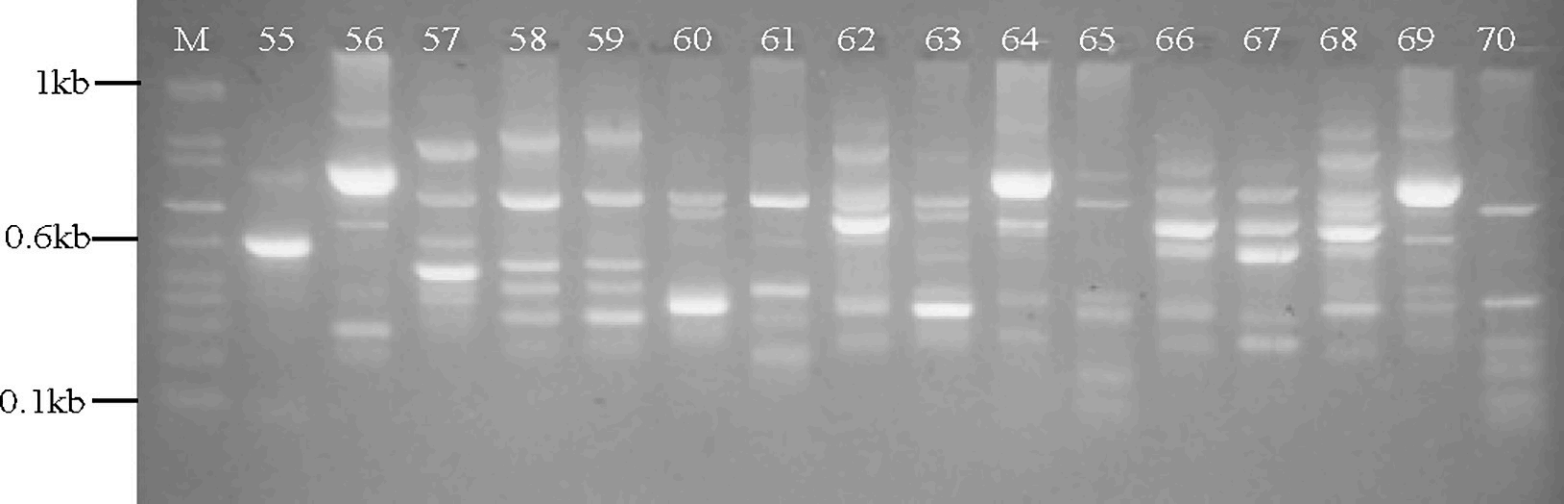
Representative example of ERIC fingerprints of different carbapenem-resistant isolates on agarose gel electrophoresis. Note: M = DNA 1 kb marker, 55 through to 70 = numbered fingerprints. By visual inspection sample numbers 58 & 59 are showing close relatedness on the gel.

The ERIC-PCR gel analysis exhibited 1 to 8 bands ranging from 50 to 800 bp. Band patterns of 93 complex dissimilarities were visually distinguished from the 111 CR isolates studied, while the remaining 18 showed band similarities in pairs. A typical gel fingerprint showing representative band patterns is shown in Fig 1.

**Fig 2:**
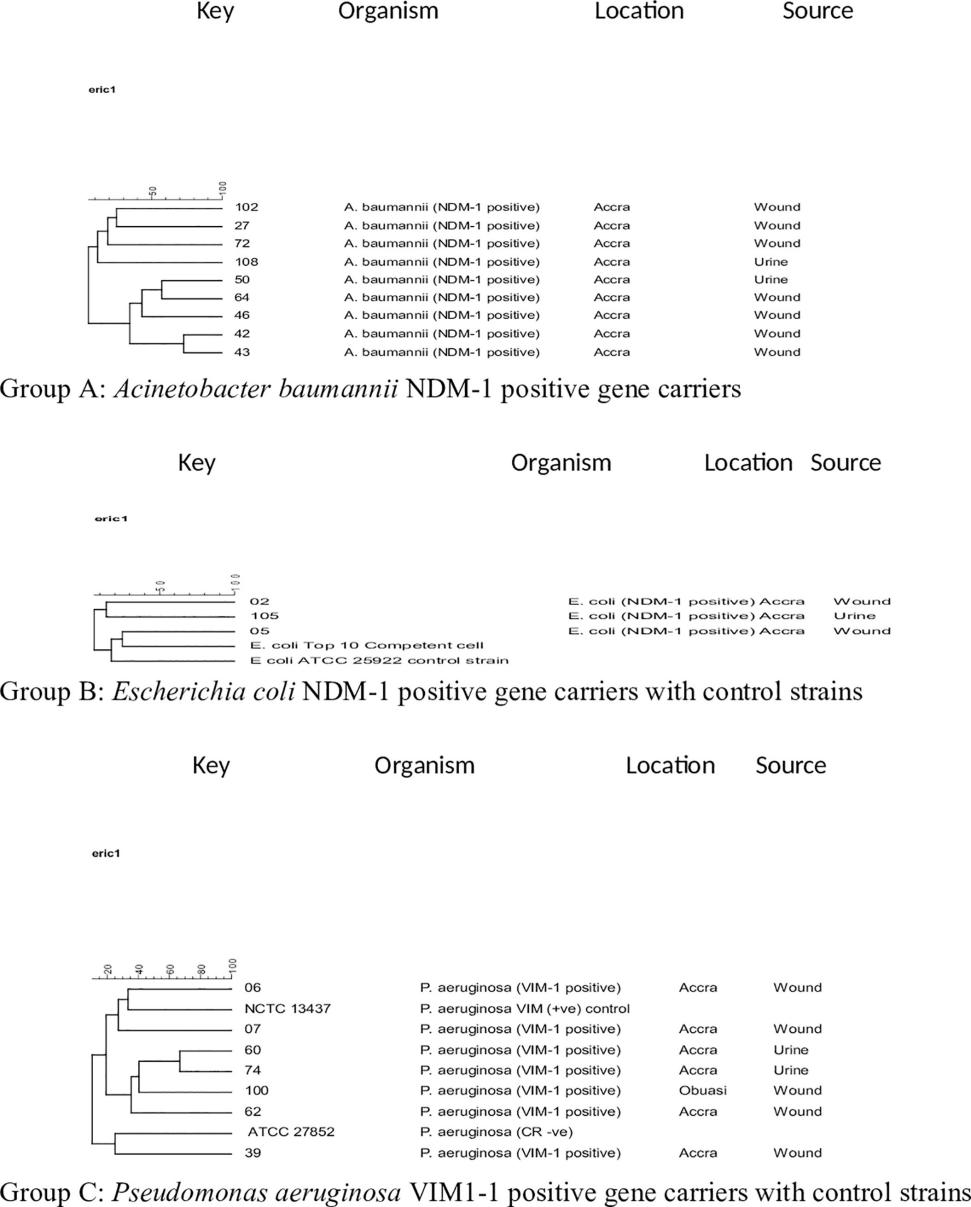
Dendrogram generated from ERIC-PCR genomic DNA products for carbapenemase-positive gene carriers. Key: isolate number or control strain/type number. Regarding the groupings based on type of species, type of resistance gene, hospital location and source of specimen, the following groupings based on type of species: Group A (*A*. *baumannii* NDM-1 positive) 102 & 27, 42 & 43 and 50 & 44 in urine and wound respectively; Group B (*E*. *coli* NDM-1 positive) 02 & 105 in wound and urine respectively; Group C (*P*. *aeruginosa* VIM-1 positive) 60 & 74 are showing close relatedness in cluster-pairs.

Dendrogram data generated from the computer-designed analysis indicated a high genetic dissimilarity among the 26 PCR-positive carbapenemase carriers with few distinguishable patterns based on species of CR isolates (Fig 2). However, 2 cluster-pairs of *Acinetobacter baumannii* and cluster-pair *Pseudomonas aeruginosa* isolates were harbouring NDM-1 and VIM-1 genes, respectively. Strikingly, the only cluster-pair of OXA-48 carrying *Klebsiella pneumoniae* isolates were genetically related from male patients, however, they were observed to have come from different age groupings, specimens and hospitals in this study. Comparatively, low numbers showed relatedness in the PCR-positive carbapenemase carriers in relation to specimen types and regional hospitals (Table 2). Overall ERIC data obtained from diverse clinical specimens indicated that there was no evidence in this study of horizontal transfer of CR isolates.

**Table 2:** Genetic relatedness among the carbapenemase-positive gene carriers.

Sample code number^a^	Name of carbapenem- resistant isolate	Regional hospitals				Type of specimen	ESBL β-lactamase genes		Carbapenemase genes		
		KBTH	ENRH	AGMH	HRH		TEM	SHV	NDM	VIM	OXA
102 & 27	*A*. *baumannii*	+	-	-	-	Wound	+	+	+	-	-
42 & 43	*A*. *baumannii*	+	-	-	-	Wound	+	+	+	-	-
50 & 64*	*A*. *baumannii*	+	-	-	-	Urine & Wound	+	+	+	-	-
02 & 105*	*E*. *coli*	+	-	-	-	Wound & Urine	+	+	+	-	-
60 & 74	*P*. *aeruginosa*	+	-	-	-	Urine	+	+	-	+	-

^a^ Cluster-paired sample numbers

* Closely related but different specimens respectively

Note: + = found in both, - = not found in both

## Discussion

This is the first study in Ghana and one of the few in sub-Saharan Africa to investigate the genetic diversity of CR Gram-negative bacilli isolates from clinical specimens. The study analysis has shown a high degree of genetic diversity among the CR isolates using the ERIC-PCR technique. ERIC-PCR fingerprints have proved the existence and expression of MBL-types namely; NDM-1- and VIM-1-type of genes following genomic DNA optimisation of the CR study isolates. A study by Abdalhamid *et al*. [22] described the expression of the two genes as highly transmissible on mobile elements that can easily spread from one patient to another in a health-care environment [22].

ERIC-PCR typing showed distinguishable fingerprints for the 111 CR isolates. In assessing the patterns of all fingerprints, 83.8% (93/111) were observed to have substantial variability among the 10 diverse CR organisms recovered from the four hospitals. However, a small number (6/82) of the non-fermenting, *Acinetobacter baumannii* and *Pseudomonas aeruginosa* isolates, were observed to exhibit close relatedness. Of significance to this study, the ERIC-PCR profiling has shown the diversity that existed among the various species of pathogens within the CR isolates. Notably, in the carbapenemase-positive gene carriers, the only two cluster-pairs, NDM-1 positive *Acinetobacter baumannii* and VIM-1 positive *Pseudomonas aeruginosa* isolates, were detected from the Korle Bu Teaching Hospital (KBTH) in the Greater Accra region and none of these cluster relations were detected in the three other regional hospitals studied. These observations imply that there is very limited transmission of resistant isolates in the four hospitals from which isolates were collected and possibly that the mobility of the resistant determinants is also low. The presence of MBL-types of resistance genes, coupled with ESBL production and the unknown number of other resistance genes encoded in the *Acinetobacter baumannii* isolates in this study is of major concern in a hospital environment [23]. The two organisms, *Pseudomonas aeruginosa* and *Acinetobacter baumannii*, have been described as environmental and opportunistic pathogens naturally adaptable in hospitals to cause serious infections, with mortality rates ranging from 18% to 61% [24]. Additionally, clonal transfer of resistance genes is commonly associated with these non-fermenting isolates in several studies worldwide [17, 25–28]. *Acinetobacter baumannii* and *Pseudomonas* species are known to cause serious, difficult to treat infections, and are ubiquitously found in the majority of health-care facilities. Many vulnerable patients, such as the elderly in ICUs, and children and babies admitted in NICU, are at an increased risk of infections caused by these organisms [29, 30]. An evidence based study carried out in paediatric and NICU wards recovered emerging carriers of OXA-type carbapenemase genes in *Pseudomonas* and *Acinetobacter* species, [14] while both species have been found as carriers of the MBL-type genes [31, 32].

Berezin *et al*. [30] described infections of *Pseudomonas* and *Acinetobacter* species as most critical when additionally associated with resistance genes for fluoroquinolones, tetracyclines, sulphonamides, and aminoglycosides encoded on the same moveable genetic elements. The presence of ESBL enzymes in these non-fermenting isolates is their common risk factors for carbapenemase resistance. Further, ESBL production becomes problematic when in association with carbapenemase resistance genes, usually identified with a reduced susceptibility to third-generation cephalosporins and quinolones. In contrast, only moderate increases in resistance to the same antimicrobial drug classes are observed in the Enterobacteriaceae group when phenotypically assessed [17, 18].

In the current study, a computer-generated ERIC-PCR profile revealed that 14.4% (16/111) of CR isolates which displayed multiple band patterns comprising; 9 (8.1%) isolates of *Acinetobacter baumannii*, 4 (3.6%) *Pseudomonas aeruginosa*, 2 (1.8%) each of *Pseudomonas putida*, *Escherichia coli* and *Enterobacter cloacae*, showed cluster patterns. These few species likely have a related origin of dissemination. A similar study conducted by Siqueira *et al*. [14] found that small pocket groupings of *Pseudomonas* and *Acinetobacter* encoded with carbapenemase resistance genes were detected showing clonal similarities by the ERIC-PCR amplification technique in a Brazilian hospital [14]. Of note, these findings were comparable to those in this present study, in which close relatedness were found in 3 cluster-pair patterns of *Acinetobacter baumannii* isolates recovered from aspirate, wound and urine, and 2 cluster-pair *Pseudomonas aeruginosa* isolates from urine and wound specimens. Interestingly, the relatedness was identified in the same hospital, Korle Bu Teaching Hospital in the Greater Accra region. The significance of the findings attest to the fact that the hospital is the largest hospital in the study, receives the largest number of patients and serves as the largest tertiary and referral centre in the whole country. However, close relatedness of ERIC-PCR fingerprints was unexpectedly observed between the 2 PCR-positive OXA-48 *Klebsiella pneumoniae* isolates since both were recovered from different hospitals, AGMH and ENRH, while the sample sites were also different, sputum and wound isolates, respectively.

The findings presented here suggest high genetic diversity existed among the CR isolates. However, these isolates may have harboured other unknown resistance genes that can potentially cause cross-transmission, together with the small number of positive NDM-1 *Acinetobacter* and VIM-1 *Pseudomonas aeruginosa* isolates identified as closely related in cluster-pair patterns by ERIC-PCR fingerprints. These resistance genes are emerging in Ghana and further infection control measures may need to be implemented in this care facility in the future to counter this threat. The significance of the genetic relatedness of the few cluster-pairs identified by ERIC-PCR has given an indication of the relatedness of carbapenemase genes in dissemination. Besides the common ESBLs (TEM-1 and SHV-1) detected, various banding patterns may be associated with other antimicrobial resistance genes. It is noteworthy and of concern, that large numbers of *Acinetobacter* and *Pseudomonas* CR isolates were negative on the PCR assay and may possibly be associated with non-carbapenemase-related resistance features or unknown resistance genes that can also disseminate into different bacterial isolates within the same health-care facility. Further studies on non-carbapenemase-related resistance need to be systematically carried out in this region.

Multiple plasmid bands in these nosocomial non-fermenting pathogens were observed in this study. These plasmids have the capacity to harbour many resistance genes, making them a clinical concern as well as a potential public health threat. The multi-resistant nature of these bacterial pathogens to commercially available antimicrobials subsequently renders their treatment extremely challenging. Of clinical significance is the emergence of plasmid-encoded AmpC cephalosporinases. These AmpC enzymes are contributors to carbapenem-resistance that may be present in those isolates of *Acinetobacter baumannii* and *Pseudomonas aeruginosa* that were negative on the carbapenemase PCR. The production of AmpC enzymes may be implicated in both carbapenemase-positive producers and CR isolates without a genetically identified locus, and may have the ability to spread to other clinically relevant pathogens in the same hospital setting.

There are a few limitations of the study. Firstly, we did not screen for all known genes that contribute to carbapenem resistance. Secondly, we could not carry out performance characteristics of the PCR assay used to test for carbapenemase genes.

In conclusion, ERIC-PCR fingerprints have shown a great diversity among the species of Gram-negative bacterial pathogens and specimen collection sites in this study. There was a small number of cluster-pairs from both carbapenemase-positive gene carriers and CR isolates without a genetically identified locus that exhibited close genetic relatedness, particularly in *Acinetobacter baumannii* and *Pseudomonas aeruginosa* isolates. These study findings underpin the need to implement stringent and preventive measures to control resistance-gene dissemination into the Ghanaian population.
